# The Treatment of Frontal Sinus Suppuration

**Published:** 1914-06-06

**Authors:** 


### MODERN METHODS SERIES.

### The Treatment of Frontal Sinus Suppuration.

For several years rhinologists have been teaching the medical student and the practitioner that the proper treatment of any intractable empyema of the frontal sinus—by no means an infrequent complaint and especially common of recent times as a sequela of influenza—is to open that cavity by cutting through first the skin in the eyebrow region, and then through the outer bony wall of the suppurating cavity. This operation, planned on the surgical principle of draining away pus wherever situated by incision into the abscess cavity, has been done now many thousands of times; and the whole medical profession has accepted the teaching of the specialists that it is the only proper thing to do when an empyema of the sinus is diagnosed and cannot be cured by non-operative measures. Various ingenious devices have been evolved for performing this operation so as to leave as little external scar afterwards as possible: these are often successful, for the regrowth of the eyebrow easily covers and hides any scar which has been made in a situation carefully chosen beforehand for this after-result.

Lately, however, some of the leading rhinologists have begun to reconsider their beliefs upon this matter. They feel, or some of them feel, dissatisfied with the results of the operation that has been classical now for some years; and they have begun to cast about for alternative methods with a view to securing improved results. At a meeting of the Laryngological Section of the Royal Society of Medicine in April last a discussion on intranasal operation was initiated by Dr. Watson-Williams and Mr. Herbert Tilley, both of whom have become converts to this method of attacking frontal sinus suppuration in preference to the external route.

### A Change of View.

The latter well-known operator "cannot escape the conclusion that more patients have died or suffered as a result of the external operation than from the untreated disease." This is a somewhat disconcerting pronouncement in view of the length of time during which the external operation has been the standard treatment; but at least it is comforting to realise that the rhinologists recognise the room for improvement and are endeavouring to secure more ideal results. Mr. Tilley makes a wise remark, too, when he asserts that many patients with chronic suppuration in the frontal sinus can get along quite well and comfortably without any operation provided they will put up with the slight inconvenience of the purulent discharge from it. Probably most rhinologists will agree concerning this, however they regard Mr. Tilley's pessimistic views of the external operation.

It is, therefore, evident that the intranasal methods which have been on trial during the last three years or so have been sufficiently successful at least to warrant exhaustive tests of their merits. Dr. Watson-Williams describes, in his communication to the Section, printed in the *Journal of Laryngology, Rhinology, and Otology*, his own technique and instruments for making an opening into the frontal sinus through the nose; and Mr. Tilley practises a procedure based upon that of Mosher, of Chicago. A perusal of these descriptions makes it clear that the intranasal operations are difficult and complex; they require a most intimate knowledge of the anatomy of the nasal passages and of the variations from the normal that may be encountered. In a word, they are for the highly expert specialist and for no one else. They require, also, a whole armamentarium of special instruments, upon the design and construction of which a vast amount of patience, ingenuity, and originality have been expended. To master the use of these specially devised tools, considerable practice must be necessary; more, in fact, than any but a specialist can possibly acquire. Dr. Watson-Williams and Mr. Tilley report between them over eighty cases, with one fatality, not due to the operation. This shows at least that in their hands the intranasal route is reasonably safe.

Mr. Tilley has now given up the external operation altogether in chronic cases; Dr. Watson-Williams still uses it sometimes, and states that "it is sometimes safer to do an external operation than to persist in intranasal methods in a wholly unsuitable case." Both authors freely grant that many cases can be cured without operation at all, or by means of comparatively minor measures, especially in acute cases. It is for those cases which go on to chronic suppuration that these major operations are required.

Mr. Tilley emphasises the shortening of the after-treatment required. In the external operation, he says, this will be rarely less than from four to six weeks, and during this period the patient is almost totally incapacitated from work or pleasure because of a bandaged or covered eye. After the intranasal operation the patient can usually go out with safety in a week. As he himself frankly says, he was one of the radical pioneers in the surgery of the frontal sinus; so that his change of view after an experience which is probably as great as that of any operator in England, is particularly significant. The time is not yet ripe for any final decision upon the relative merits of these two ways of treating frontal empyema which does not respond to less radical measures; but it seems quite possible that in a few years this new method may have entirely superseded the old one.

As regards acute suppuration in the sinus, intranasal methods have always been recognised as efficacious and preferable in the first instance to external methods: so the change of practice which Dr. Watson-Williams and Mr. Tilley advocate applies much less to this particular form of the mischief.

